# Identification of immunological subtypes of hepatocellular carcinoma with expression profiling of immune-modulating genes

**DOI:** 10.18632/aging.103395

**Published:** 2020-06-16

**Authors:** Di Cao, Meng-Ke Chen, Qing-Feng Zhang, Yu-Feng Zhou, Mei-Yin Zhang, Shi-Juan Mai, Yao-Jun Zhang, Min-Shan Chen, Xiao-Xing Li, Hui-Yun Wang

**Affiliations:** 1 State Key Laboratory of Oncology in South China, Collaborative Innovation Center for Cancer Medicine, Sun Yat-Sen University Cancer Center, Guangzhou, China; 2 Department of Hepatobiliary Oncology, Sun Yat-Sen University Cancer Center, Guangzhou, China; 3 Precision Medicine Institute, The First Affiliated Hospital, Sun Yat-Sen University, Guangzhou, China

**Keywords:** hepatocellular carcinoma, immune checkpoint, B7-H3, CD47, CD8

## Abstract

Recent studies demonstrate that immune checkpoint inhibitor (ICI) therapy has achieved success in many types of advanced cancers including advanced hepatocellular carcinoma (HCC). However, ICI therapy is beneficial in only some HCC patients, suggesting that immune-responses are highly variable in HCCs. Therefore, understanding the immune status in HCC microenvironment will facilitate ICI immunotherapy and guide patient selection for the therapy. In this study, we first analyzed the expression profile of immune-modulating genes and their relationship with survival of HCC patients using the data downloaded from The Cancer Genome Atlas - Liver Hepatocellular Carcinoma (TCGA-LIHC) database, and found that the higher expressions of CD276 (B7-H3) and CD47 were significantly associated with poor survival. Then we identified 4 immune subtypes of HCCs with different survivals by using the combination expression of B7-H3 (or CD47) and CD8. Patients with B7-H3^low^/CD8^high^ or CD47^low^/CD8^high^ have the best survival while ones with B7-H3^high^/CD8^low^ or CD47^high^/CD8^low^ have the worst survival. The 4 immune subtypes were validated in another 72 HCC patients obtained from South China. In conclusion, our findings suggest that HCC patient prognosis is associated with immunophenotypes by T cell infiltration (CD8 expression) and the expression of the adaptive immune resistance gene (B7-H3 or CD47), and this immune classification system will facilitate HCC patient selection for ICI immunotherapy.

## INTRODUCTION

Hepatocellular carcinoma (HCC) is a malignant liver tumor characterized by very poor survival, and is the second leading cause of cancer death worldwide [1]. One important reason for HCC poor survival is the limited effect of chemotherapy, radiotherapy, and surgical resection can only be performed in patients with early stage HCC [2]. Recent reports show that immunotherapy is a highly promising therapeutic method in many advanced cancers, particularly in those induced by viruses [3, 4]. In China, most HCC patients are infected with hepatitis B virus and have chronic hepatitis, indicating that HCC patients may be suitable for immunotherapy. More important, the liver is recognized as a critical site for the development of immune tolerance to cancer [5], where cancer cells are not sensed and destroyed by the immunosystem [6]. In addition, one study has shown that dysregulation of hepatic immunotolerance is involved in liver carcinogenesis and progression [7]. Therefore, immunostimulatory treatment is potentially effective for HCC patients, and may help restore anti-cancer immunity and prolong the survival of these patients.

Recently, studies demonstrate that immune checkpoint molecules (also called immunomodulators), which are pathways that balance the immune response and protect the host from autoimmunity, are dysregulated in the tumor microenvironment (TME) of many cancers [8]. The immune checkpoint molecules include programmed cell death protein 1 (PD-1), cytotoxic T lymphocyte–associated antigen 4 (CTLA-4), PD-1 ligand 1 (PD-L1), B7 homolog 3 (B7-H3) and others. When dysregulated in the TME, the immune checkpoint molecules could suppress anti-tumor immune responses in many cancers including liver cancer, resulting in the development and progression of cancer. Therefore, these inhibitory immune checkpoint molecules have become targets for immunotherapy, and now seven immune checkpoint inhibitors (ICIs) have been developed and approved by US Food and Drug Administration for the treatment of advanced cancer [https://medi-paper.com/us-fda-approved-immune-checkpoint-inhibitors-approved-immunotherapies/]. However, most of the ICIs are effective in only a portion of patients. For example, Nivolumab and Pembrolizumab (antibodies against PD-1) have approximately 16–20% of objective response rate in unselected patients with advanced HCC [9, 10], which implies that there is a great need for understanding the immunologic characteristics of HCCs for optimal selection of patients who may respond to immune-based therapies [11–15].

In 2017, Sia D et al found 25% of HCC patients have high-expression of identified immune checkpoints and cytolytic activity markers, suggesting that these patients might be sensitive to ICI therapy [16]. Kim et al subsequently identified two subgroups of HCCs: the first group with a discrete population of PD-1^high^ CD8^+^ T-cells was more aggressive, and the second group without a discrete population of PD-1^high^ CD8^+^ T-cells was much less aggressive; the first HCC group had higher levels of predictive biomarkers of response to anti-PD-1 therapy, and incubation of the T cells from these HCCs with antibodies against PD-1, TIM3 or LAG3 restored proliferation and production of IFNγ and TNF in response to anti-CD3 treatment [17, 18]. These findings clearly indicate that the efficacy of immune checkpoint-based therapy is mainly related to T cell antitumor activity in the TME but not the tumor cell phenotype [19]. Therefore, elucidation of the subtypes of infiltrated T cells in HCC tissues will facilitate personalizing ICI therapy and potentially prolong the survival of HCC patients.

In this study, we first utilized the clinical survival and RNA expression data downloaded from The Cancer Genome Atlas - Liver Hepatocellular Carcinoma (TCGA-LIHC) database to analyze the relationship between the immune-checkpoint gene expression profile and survival of patients with HCC, and identified additional dysregulated immune checkpoints that may be implicated in tumor progression. Then, we confirmed the clinical significance of these dysregulated immune checkpoints using immunohistochemistry (IHC)-based method in an HCC patient cohort obtained from Sun Yat-Sen University Cancer Center. Our findings provide broad understanding of HCC immunophenotypes and indicate opportunities for establishing systemic immunotherapies in HCC.

## RESULTS

### The expression patterns and clinical significance of immunomodulators in HCC patients

**Figure 1:**
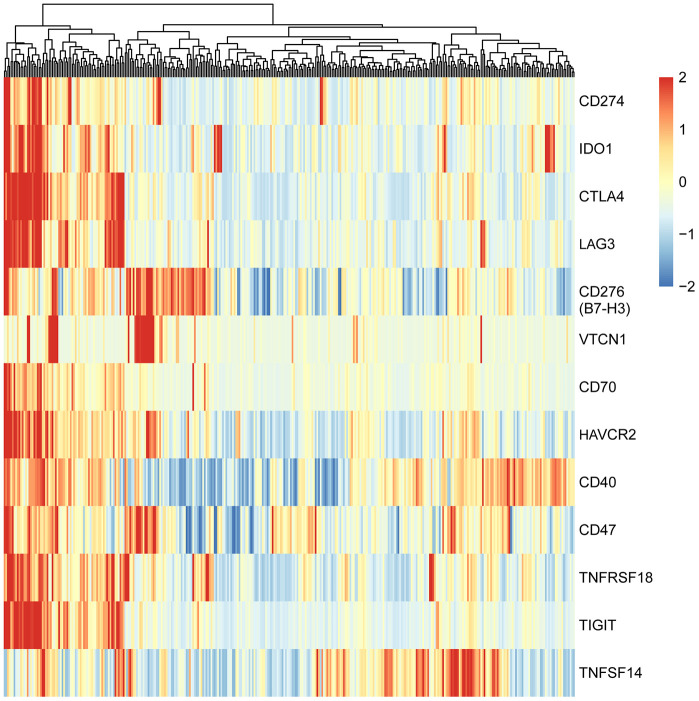
**Heatmap of adaptive immune resistance genes in HCC patients of TCGA-LIHC cohort.** The data were downloaded from TCGA-LIHC dataset and analyzed with clustering method. All HCC patients (374 cases) were clustered into two groups: one (73 cases) was characterized by high-expression of nearly all adaptive immune resistance genes, and the other (301 cases) by low-expression of the genes. Red bar represents gene high expression, and green represents gene low expression. Each column indicates one sample.

**Figure 2:**
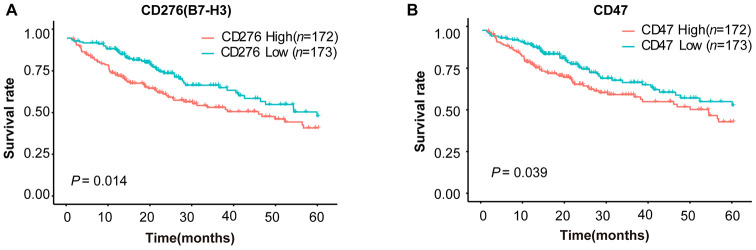
**Survival curves of HCC patients stratified by B7-H3 (CD276) or CD47 expression levels in TCGA-LIHC cohort.** HCC patients (*n* = 345) were divided into high or low expression group based on the median value of gene expression. (**A**) Survival curves of HCC patients with high and low B7-H3 expressions. (**B**) Survival curves of HCC patients with high and low CD47 expressions. The *P*-value cut-off was 0.05 (log rank test).

To explore the expression patterns of immunomodulators in HCC, we performed clustering analysis of expression of immune-modulating pathway genes, in which RNA-seq data were downloaded from TCGA-LIHC database. A group of selected immune checkpoint genes were upregulated mainly in a subset of HCC tissues, implying that these immune checkpoint genes might be activated in only a small portion of HCC patients (Figure 1). To elucidate the clinical significance of the upregulated immune checkpoints, we analyzed the relationship between these genes and overall survival (OS) of HCC patients. The results show that only two genes are significantly associated with poor OS: CD276 (B7-H3) (*P* = 0.014, Figure 2A) and CD47 (*P* = 0.039, Figure 2B), consistent with previous reports [20–25]. All the others do not have a statistically significant relationship with survival (Supplementary Figure 1). These results suggest that the upregulated B7-H3 and CD47 may lead to immunosuppression, resulting in poor survival in HCC patients.

High tumor-infiltrating lymphocyte density in TME is often associated with better survival in many tumor types [26, 27]. However, there is no significant relationship between the survival and the expression of any canonical lymphocyte subset markers including CD4, CD8, or CD19 in HCC patients (all, *P*> 0.05, Supplementary Figure 2), based on the mRNA expression from TCGA-LIHC data. These results imply that the number of infiltrated lymphocytes may be small in most of the HCC tissues or big in only a small part of tumor tissues so that these lymphocyte markers could not relay the statistical significance in the survival analysis for the whole cohort of HCC patients.

Given that high expression of B7-H3 and CD47 is associated with poor prognosis in HCC, we subsequently investigated their relationship with other clinical factors. Recently, tumor mutational burden (TMB) is reported to be related with the effectiveness of ICI immunotherapy [28], and an average of 30–40 mutations per tumor are identified in HCC, which represents a high TMB [29]. A high TMB may produce more neoantigens that can induce the infiltration of neoantigen-specific CD8^+^ lymphocytes into the tumor tissues, indicating a possible upregulation of the adaptive immune resistance genes in the T cells or tumor cells_._ Therefore, we analyzed the relationship between B7-H3 or CD47 expression and TMB in HCC patients. However, the results show no significant relationship between the TMB and B7-H3 or CD47 expression in TCGA-LIHC database (Supplementary Figure 3A, 3B).

Then we explored the relationship between the expression of these molecules and other clinical features. As shown in Supplementary Figure 4, the increased B7-H3 expression is positively correlated with increased tumor stage, and increased CD47 expression is marginally correlated with increased tumor stage. All of these results suggest that the upregulated immune checkpoints B7-H3 and CD47 may increase the development and progression of HCC.

### B7-H3 and CD47 expressions are positively correlated with subsets of infiltrated T cells in HCC

**Figure 3:**
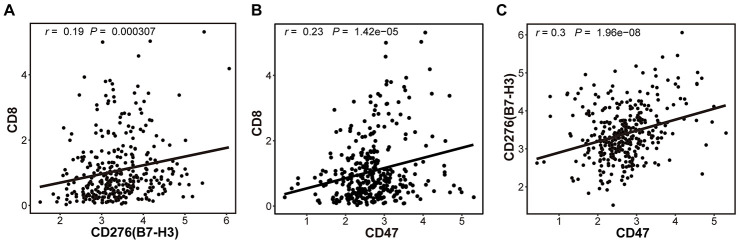
**The correlations between 3 immune markers (B7-H3, CD47 and CD8) in TCGA-LIHC cohort.** The correlations between the expressions of CD8 and B7-H3 (**A**), CD8 and CD47 (**B**) and B7-H3 and CD47 (**C**), were presented with Scatter plots (based on 345 HCC samples), and there are significantly positive correlations between 3 pairs of immune markers. The Pearson correlation coefficient (r) and corresponding *P*-value are shown in each plot. The *P*-value cutoff was 0.05.

One study indicates that CD8^+^ cytotoxic T cells in the TME can upregulate the adaptive immune resistance genes via enhancing the expression of IFNγ [30, 31]. In this study, though the mRNA was originated from HCC tissues including HCC cells, infiltrated lymphocyte cells and other mesenchymal cells, the amount of CD8 mRNA mainly represented the number of infiltrated T cells because CD8 was principally expressed on T cells. Therefore, we explored whether CD8 expression was associated with B7-H3 or CD47 expression (Figure 3). As expected, a positive relationship between CD8 and B7-H3 (*r* = 0.19, *P* = 3.07 × 10^-4^) or CD8 and CD47 (*r* = 0.23, *P* = 1.42 × 10^-5^) was observed (Figure 3A and 3B), indicating that the increased CD8^+^ T cells may have upregulated the adaptive immune resistant genes by activating IFNγ pathway in HCC patients. In addition, B7-H3 and CD47 expressions were also observed to be positively correlated with each other (*r* = 0.30, *P* =1.96 × 10^-8^, Figure 3C).

**Figure 4:**
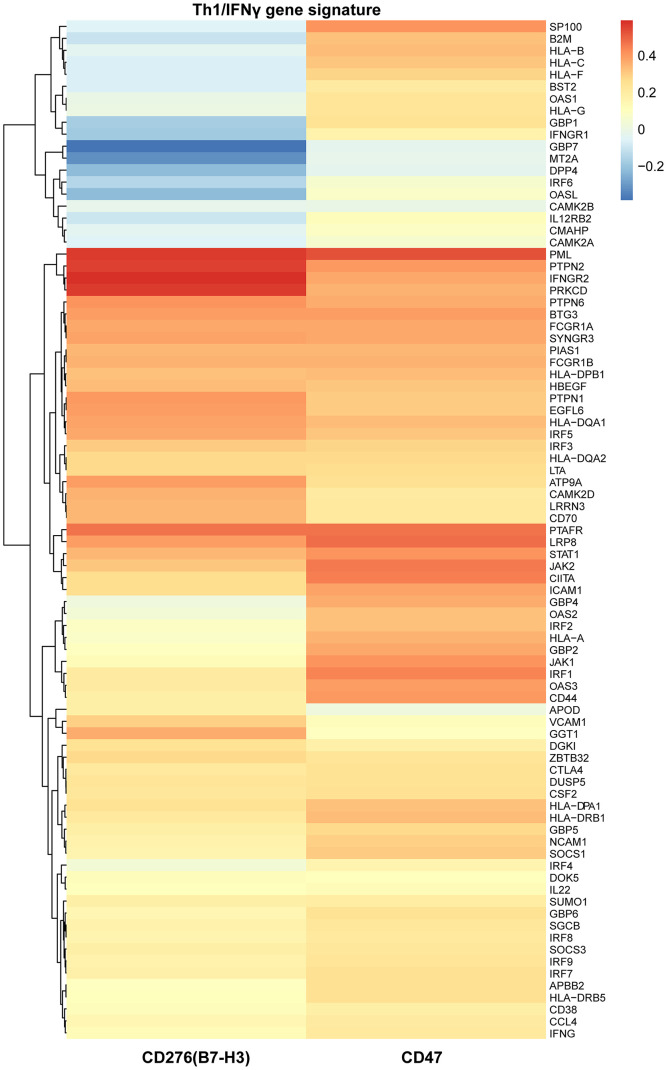
**The relationships of B7-H3 and CD47 with Th1/IFNγ gene signature in TCGA-LIHC cohort.** Heat map shows the correlations of each gene expression of Th1/IFNγ gene signature with B7-H3 and CD47 expression, which were computed based on 345 HCC cases. Red color indicates positive Pearson correlation coefficients; blue color indicates negative correlations.

Similarly, T helper type 1 (Th1) cells also produce IFNγ and subsequently activate IFNγ gene signature which is primarily responsible for activating an anti-tumor immune response including an increase of infiltrated cytotoxic T cells in the TME. Therefore, we supposed that IFNγ gene signature might be correlated with B7-H3 or CD47 expression. To this end, we performed a correlation analysis of Th1/IFNγ gene signature with B7-H3 or CD47 expression. As shown in Figure 4, most genes of Th1/IFNγ gene signature have a positive correlation with B7-H3 or CD47 expression. These results imply that the infiltration of CD8^+^ T cells and Th1 cells may increase the expression of B7-H3 and CD47, or increased expression may enhance cytotoxic T cell infiltration and Th1 differentiation.

### Identification of HCC immune subtypes with different survivals

**Figure 5:**
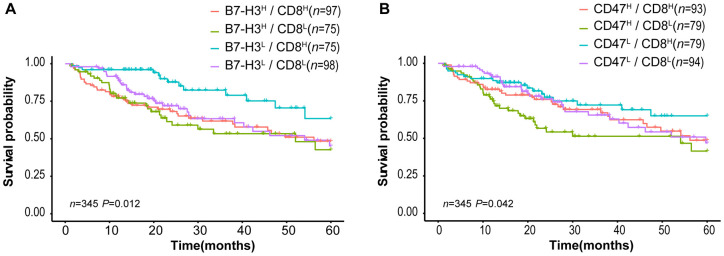
**Survival curves of HCC patients stratified by combined CD8 expression and B7-H3 or CD47 expression in TCGA-LIHC cohort.** (**A**) Overall survival curves of HCC patients stratified by the combined expressions of B7-H3 (CD276) and CD8: the survival of patients with B7-H3^low^/CD8^high^ is significantly better than that of those with B7-H3^high^/CD8^high^ (*P* = 0.004), B7-H3^high^/CD8^low^ (*P* = 0.001) or B7-H3^low^/CD8^low^ (*P* = 0.012) and no significance between other groups. (**B**) Survival curves of HCC patients stratified by the combined expressions of CD47 and CD8: the survival of patients with CD47^high^/CD8^low^ is significantly worse than that of those with CD47^low^/CD8^high^ (*P* = 0.008) or CD47^low^/CD8^low^ (*P* = 0.045), and no significance between other groups (log rank test). H represents high and L represents low respectively.

Tumor-infiltrating CD8^+^ T lymphocytes are associated with favorable survival in multiple tumor types [32] while B7-H3 or CD47 expression is correlated with poor survival of cancer patients. In our results described above, different HCC patients have distinct expression patterns of CD8 and B7-H3/CD47, although CD8 expression is positively correlated with B7-H3 or CD47 expression (Fig 3). Therefore, we supposed that HCC patients with different patterns of CD8 and B7-H3/CD47 have different survivals. To confirm this, we first divided HCC patients into 4 immune subtypes based on the expression patterns of CD8 and B7-H3 in HCC tissues: B7-H3^high^/CD8^high^, B7-H3^low^/CD8^high^, B7-H3^high^/CD8^low^, and B7-H3^low^/CD8^low^. Then we performed survival analysis on the patients with 4 immune subtypes. As expected, patients with B7-H3^low^/CD8^high^ had the best outcome, whereas patients with B7-H3^high^ /CD8^low^ had the worst outcome. Similar results were obtained from the patients stratified by the expression patterns of CD8 and CD47 (Figure 5B). These results suggest that the combined analysis of CD8 and B7-H3 (or CD47) expression can identify different HCC immune subtypes that have distinct survivals.

### HCC immune subtypes are validated in patients from South China

**Figure 6:**
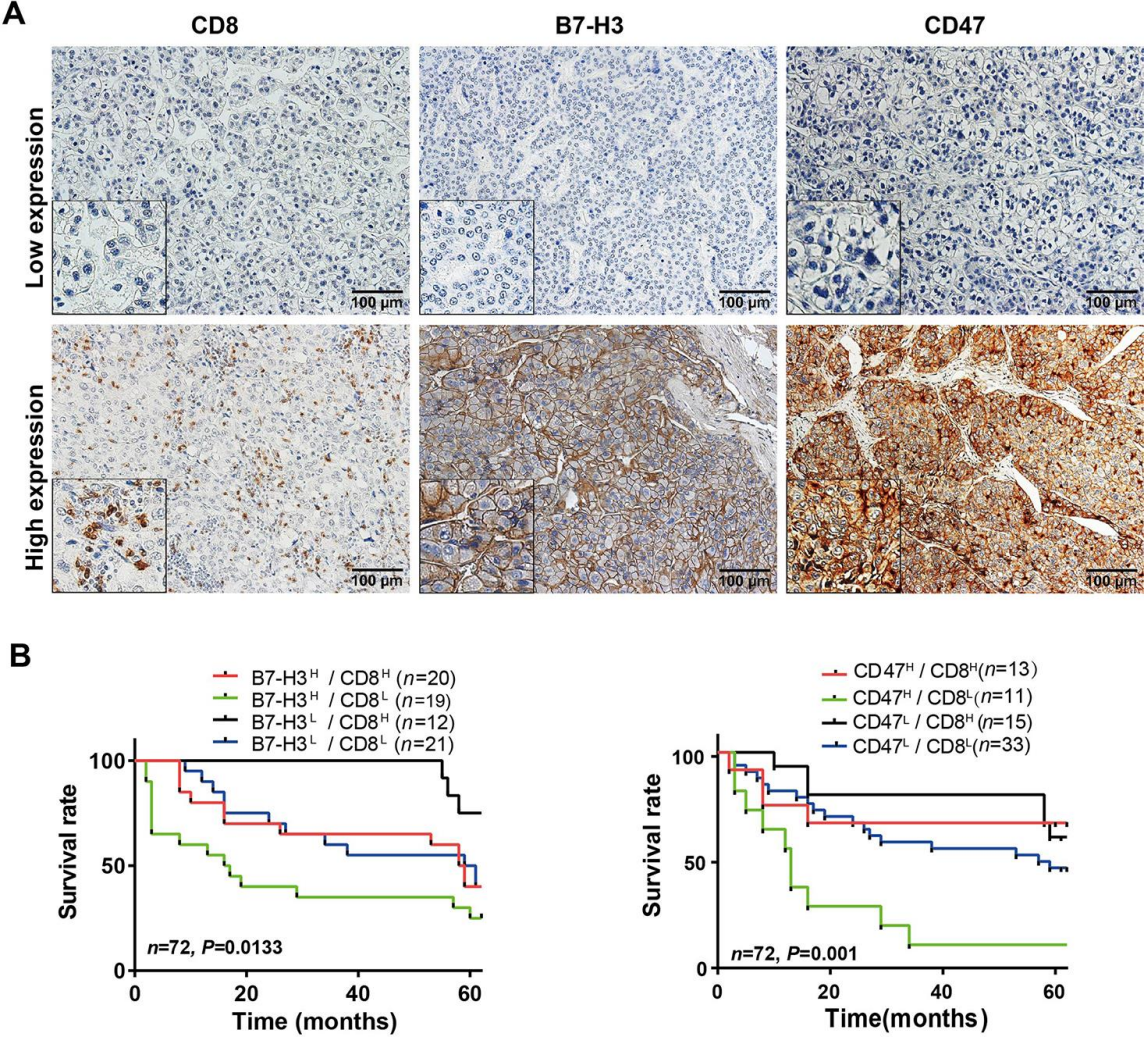
**Parallel analysis of the effect of B7-H3, CD47 and CD8 on survival of HCC patients in the SYSUCC cohort.** (**A**) Representative images of high and low expression of CD8, B7-H3 and CD47 detected by IHC in HCC tissues (Magnifications: 200× and 400x [the left-lower corner in the images]). (**B**) Survival curves of HCC patients stratified by combined expressions of B7-H3 and CD8 proteins: the survival of patients with B7-H3^low^/CD8^high^ is significantly better than that of those with B7-H3^high^/CD8^high^ (*P* = 0.043), B7-H3^high^CD8^low^ (*P* = 0.003) or B7-H3^low^/CD8^low^ (*P* = 0.048) and no significance between other groups. (**C**) Survival curves of HCC patients stratified by combined expression of CD47 and CD8 proteins: the survival of patients with CD47^high^/CD8^low^ is significantly worse than that of those with CD47^high^/CD8^high^ (*P* = 0.01), CD47^low^CD8^high^ (*P* < 0.001) or CD47^low^/CD8^low^ (*P* = 0.003) and no significance between other groups. H represents high and L represents low respectively (log rank test).

To validate the HCC immune subtypes identified from the mRNA expression data of TCGA-LIHC database, we first collected 72 HCC samples from SYSUCC, in South China and detected the protein levels of CD8 and B7-H3/CD47 in HCC tissues by IHC. The IHC scoring of CD8 expression on immune cells and B7-H3 or CD47 on tumor cells was performed by two experienced pathologists who were blinded to the patients’ clinical information. The representative IHC images of B7-H3, CD47 and CD8 were presented in Figure 6A. Then we analyzed the relationships of B7-H3 and CD47 expression with CD8 expression and clinical characteristics. As shown in Table 1, B7-H3 or CD47 expression is significantly positively correlated with CD8 level, but there is no significant correlation with other clinical characteristics, including sex, age, tumor number, tumor size, differentiation grade and clinical stage (all comparisons, *P*> 0.05). Next, we conducted survival analysis on the IHC scoring of CD8, B7-H3 and CD47 expression. According to the IHC scoring on CD8 and B7-H3, HCC patients were separated into four groups: B7-H3^low^/CD8^low^, B7-H3^low^/CD8^high^, B7-H3^high^/CD8^low^, and B7-H3^high^/CD8^high^. The survival analysis demonstrated that B7-H3^low^/CD8^high^ group had the best survival, whereas B7-H3^high^/CD8^low^ patients had the worst survival (*P*< 0.001, Figure 6B), which was consistent with the results obtained from TCGA-LIHC data. Finally, we performed the same analysis on CD47 and CD8 expression, and acquired the similar result: CD47^low^/CD8^high^ subtype patients had the best survival and CD47^high^/CD8^low^ subtype ones had the worst survival (*P*< 0.001, Figure 6C). These results indicate that we have identified 4 immune subtypes for HCC patients that have distinct survivals, which will provide a new tool for the personalized immunotherapy in HCC.

**Table 1:** Association of B7-H3 and CD47 expression with clinical characteristics and CD8^+^ T cell infiltration in patients with HCC.

**Characteristic**	**Tumor B7-H3 Density/HPF#**			***P* value**	Tumor CD47 Density/HPF*		***P* value**
	**cases**	**Low**	**High**		**Low**	**High**	
	***N***	***N* (%)**	***N* (%)**		***N* (%)**	***N* (%)**	
**Gender**							
Female	11	4 (13.79)	7 (16.28)	1	8 (19.51)	3 (9.68)	0.413
Male	61	25 (86.21)	36 (83.72)		33 (80.49)	28 (90.32)	
**Age**							
<45	36	12 (41.38)	24 (55.81)	0.23	20 (48.78)	16 (51.61)	0.812
≥45	36	17 (58.62)	19 (44.19)		21 (51.22)	15 (48.39)	
**Tumor number**							
1	53	21 (72.41)	32 (74.42)	0.85	30 (73.17)	23 (74.19)	0.922
≥2	19	8 (27.59)	11 (25.58)		11 (26.83)	8 (25.81)	
**Tumor size**							
<1cm	48	16 (55.17)	32 (74.42)	0.089	26 (63.41)	22 (70.97)	0.501
≥1cm	24	13 (44.83)	11 (25.58)		15 (36.59)	9 (29.03)	
**Pathological Grade**							
Low/Moderate	41	17 (58.62)	24 (55.81)	0.841	27 (65.85)	14 (45.16)	0.079
High	31	12 (41.38)	19 (44.19)		14 (34.15)	17 (54.84)	
**Clinical Stage**							
stage I~II	50	20 (68.97)	30 (69.77)	0.942	32 (78.05)	18 (58.07)	0.068
stage III~IV	22	9 (31.03)	13 (30.23)		9 (21.95)	13 (41.93)	
**CD8 Expression**							
Low <10	43	22 (75.86)	21 (48.84)	0.0283	30 (73.17)	13 (41.93)	0.007
High ≥10	29	7 (24.14)	22 (51.16)		11 (26.83)	18 (58.07)	

Note: ^#^ Tumor B7-H3 Density/HPF: <10% of tumor cells as low expression, ≥10% of tumor cells as high expression. *Tumor CD47 Density/HPF: <1% of tumor cells as low expression, ≥1% of tumor cells as high expression.

## DISCUSSION

Although ICIs have shown substantial treatment success in some cancers, they have inadequate effectiveness in others, and only a few patients respond to these therapies [19]. Enhanced understanding of T cell exhaustion in the TME is needed to overcome the limitations of these therapies. It is widely accepted that successful anti-tumor immune responses following PD-1/PD-L1 blockade require tumor-specific T cell reactivation and clonal proliferation in the TME, and the different results of cancer immunotherapy can be partly ascribed to the TME heterogeneity [33, 34]. Most current studies on the determinants of clinical responses to ICIs have focused on tumor intrinsic factors, e.g., tumoral PD-L1 expression or oncogenic mutational load [35, 36] instead of characteristics of tumor-infiltrating T cells. The CD8^+^ T cells in the TME are the main effectors and cytotoxic T cells against tumor cells. Thus, characterization of tumor-infiltrating CD8^+^ T cell and its exhaustion will aid not only the identification of patients with different treatment responses, but also the determination of the personalized treatment strategies.

In the present study, we utilized TCGA-LIHC data to systematically analyze the expression of immune regulatory molecules and their clinical significance in HCC, and identified four immune subtypes with distinct survivals. The four immune subtypes were validated by IHC in 72 HCC patients from South China. The results have shown the following: (i) there is an association between high B7-H3 expression and poor survival in HCC, which is consistent with previous reports [21–24]; (ii) B7-H3 expression is positively associated with CD8 expression and a Th1/IFNγ gene signature in HCC; and (iii) patients with B7-H3^low^/CD8^high^ subtype are correlated with favorable survival in HCCs. Our findings support the view that, despite the limited efficiency of immunotherapy, the endogenous anti-tumor immune response is a critical factor for survival in HCCs. Specifically, our results imply that the combined analysis of B7-H3 and CD8 expression levels may yield a better assessment of the immunologic state of HCCs and patient survival than either B7-H3 or CD8 expression alone. Our findings also provide a possibility for developing immunotherapeutic approaches based on the HCC immunologic subtypes.

B7-H3 is expressed in various tumor cells and tissues, and its high expression has been associated with poor prognosis in various cancers, including HCC [21–24]. However, these studies have only focus on B7-H3 expression and its relationship with survival, and none of them has analyzed both B7-H3 and CD8 expressions simultaneously. Despite B7-H3 was originally characterized as a stimulator of T cells [37], growing evidence has indicted it may indeed be an inhibitor [37–40]. In human HCC, B7-H3 dominantly functions in a inhibitory manner in tumor immunity via decreasing T cell proliferation and IFNγ production and B7-H3 can also be induced as a consequence of an anti-tumor T cell response, suggesting an adaptive immune resistance [23, 24]. Accordingly, the positive correlation between B7-H3 expression and CD8 expression or Th1/IFNγ gene signature in our study implies that compensatory B7-H3 upregulation in HCC is induced as a result of an anti-tumor T cell response and patients with different expression patterns of B7-H3/CD8 have significantly different survivals. Therefore, our study suggests that the personalized immunotherapy should be based on both B7-H3 and CD8 expressions.

HCC is a heterogeneous group of tumors with diversified immune characteristics. So far, ICIs have failed to demonstrate a clinical benefit in most HCCs due to its extensive immune heterogeneity. Therefore, there is a need to improve the assessment of the HCC immunologic state for the selection of subsets of patients who may benefit clinically from the immunotherapies. B7-H3 blockade enhances anti-tumor immune activity in preclinical models and early phase clinical trials [41, 42]. An anti B7-H3 antibody, enoblituzumab (also referred to as MGA271) has been shown to produce antitumor responses in a fraction of tumor patients in a recent phase I clinical trial [43]. In our study, the subset of HCC patients with B7-H3^high^/CD8^high^ may have the greatest benefit from B7-H3 inhibitor therapy as it may reactivate the antitumor response of CD8^+^ T cells and prolong the survival of patients. Our results are consistent with a recent integrated study on HCC, which identified that approximately 25% of HCC patients belong to an “Immune class” characterized by enrichment of immune cell infiltration, immune checkpoint expression, and active IFNγ signaling [16], greatly resembling that of the most immunotherapy-responsive cancers [44–46]. In this “Immune class”, patients could be divided into active and exhausted immune subtype, and the active immune subtype patients would respond to ICI therapy and had a favorable prognosis. This finding can explain why only a portion of HCC patients respond to ICIs, which underscores the significance of optimizing patient selection in immunotherapy.

In this study, we also identified that the elevated CD47 expression is correlated with poor survival in HCCs, which is consistent with previous observations [25, 47–51]. In 2015, William C Chapman et al. have shown that blocking CD47 with specific antibodies has therapeutic efficacy in human HCC [52]. CD47 expression on tumor cells can bind to its counter-receptor signal regulatory protein alpha (SIRPα) on macrophages and produces a “don’t eat me” signal [53], thus serving as an innate immune checkpoint to inhibit phagocytosis of tumor cells and tumor antigen-presenting to T cells [54–56]. Recently, CD47 was also reported to be a marker of tumor-initiating cells in various cancer types including HCC, which is responsible for a higher capacity in tumorigenicity, progression and metastasis [57–60]. In addition, CD47 expression on T cells interacts with thrombospondin-1 (TSP-1), thus acting as an adaptive immune checkpoint to inhibit T cell activation [61–63] and blockade or loss of CD47 signaling in T cells is sufficient to stimulate T cell cytotoxicity against tumors [64]. Thus, blocking CD47 could enhance anti-tumor innate immunity mediated by macrophages and anti-tumor adaptive immunity indirectly via suppressing immunosuppressive signals in antigen presenting cells [65, 66] and directly via activating T cell cytotoxicity. Our study has shown that patients with different expression patterns of CD47/CD8 have significantly different survivals, suggesting the personalized immunotherapy should be developed based on the combination of CD47 and CD8 expression, which is consistent with the report that the therapeutic effects of anti-CD47 antibody were abrogated in CD8^+^ T cell-deficient mice [54]. Another finding of our study was that a subset of patients highly expressed more than one adaptive immune-resistance molecules, which implies that multiple ICIs can be combined for the immunotherapy on the basis of immune biomarkers in the TME. A recent study showed a combination of anti-PD-1, anti-CTLA4, and anti-CD47 antibodies together has extended survival compared with anti-PD-1 and anti-CTLA4 alone or anti-CD47 alone in a syngeneic mouse model of esophageal squamous carcinoma [67]. A phase Ib clinical trial showed that rituximab in combination with 5F9, a macrophage checkpoint inhibitor that targets CD47, resulted in increased antitumor activity in patients with aggressive and indolent lymphoma [68]. However, currently there is a lack of clinical report that it can improve outcomes in HCCs.

Despite the limitations inherent in the present retrospective study, i.e., small number of specimens and sample selection biases in SYSUCC cohort, we analyzed two different patient cohorts with well-documented clinical information and survival data and found that HCC patients can be divided into 4 immune subtypes with different survivals, in which B7-H3^low^/CD8^high^ or CD47^low^/CD8^high^ subtype patients have the best survival and B7-H3^high^/CD8^low^ or CD47^high^/CD8^low^ subtype ones have the worst survival. These findings support the theory that an active anti-tumor microenvironment can predict long-term survival in HCCs and provide clues for establishing personalized immunotherapy with optimal patient selection based on the combined biomarkers. Finally, our findings warrant future study on immune subtypes and immunotherapy in larger HCC cohorts.

## MATERIALS AND METHODS

### TCGA data

The RNA sequencing (RNA-seq) level 3 data and FPKM (fragments per kilobase of exon model per million mapped reads)-normalized data in TCGA LIHC (Liver HCC, *n* = 374) database were first downloaded from the UCSC Xena website (https://xena.ucsc.edu). Then somatic mutation annotation files for patients with HCC (*n* = 364) were downloaded from the same website, and R package maftools was used to compute the tumor mutational burden. Finally, survival data were also downloaded from the website, and 29 cases were deleted from the further survival analysis because the follow-up days for these patients were less than one month. The data were analyzed with custom routines and built-in packages of R/Bioconductor software (version 3.5.0).

### HCC patients

In this study, we collected 72 HCC specimens that were randomly selected from The Tissue Bank of Sun Yat-Sen University Cancer Center (SYSUCC). All of the 72 HCC patients from whom the HCC samples were obtained had undergone surgical resection between 2005 and 2008 at SYSUCC. The patients were diagnosed as HCC pathologically and did not receive any other treatment before operation. The clinical characteristics of the patients were listed in Table 2. The clinical staging was defined according to tumor-node-metastasis staging system of the 7^th^ Union for International Cancer Control/American Joint Committee on Cancer. All patients included in the study had detailed follow-up and long-term survival data. Overall survival (OS) is defined as time from surgical resection to death or the last follow-up. This study was approved by the Ethics Committee of SYSUCC. Written informed consents were obtained from every patient prior to surgery.

**Table 2:** Clinical characteristics of HCC cases included in SYSUCC cohort (*N*=72).

**Patient characteristics**	***N***
Age at surgery (Mean)	
<45	36
≥45	36
Gender	
Female	11
Male	61
Tumor number	
1	53
2	15
3	3
4	1
Tumor size(diameter)	
<1cm	45
1~2cm	17
>2cm	10
Clinical Stage	
I	32
II	18
III	7
IV	15
Differentiation grade	
Low	3
Moderate	38
High	31
Outcomes	
Short-survivors (<18 m)	25
Long-survivors (≥18 m)	47

### IHC staining

Consecutive sections (5-μm thick) were cut from the paraffin-embedded tissues and mounted on the glass slides for IHC analysis. All HCC tissues were diagnosed by two experienced pathologists [69]. The tumor histological grading was performed according to the Edmondson grading system. The HCC tissue sections were detected by IHC staining according to standard protocols. In brief, the slides were first deparaffinized, rehydrated, and washed. Then antigen retrieval was performed in microwave oven, and the slides were incubated with 0.3% hydrogen peroxide to inhibit endogenous peroxidase activity and goat serum to block nonspecific binding sites. Next, we incubated the sections overnight with rabbit monoclonal antibody (mAb) against human B7-H3 (1:300, Cell Signaling Technology Inc.), human CD47 (1:300, Cell Signaling Technology Inc.), or human CD8 (1:200, Cell Signaling Technology Inc.) at 4°C. After serially washing, the slides were incubated with Dako REAL™ EnVision™ secondary antibody. Finally, the slides were counterstained with hematoxylin. Supplementary Table 1 lists the additional staining reagent information for CD8, B7-H3, and CD47.

To assess the tumor infiltration of CD8^+^ T cells, we counted positive-staining cells manually in five separate fields in the tumor compartment under ×200 high-power field (HPF). To define the high- or low-infiltration of CD8^+^ T cells, we used cut-off of 10 cells/HPF for CD8^+^ cells. For quantifying B7-H3 and CD47 expressions in tumor cells, we employed the proportion of cells with B7-H3 or CD47 positive staining to the total tumor cells, and high- or low-expression was determined by a cut-off value of 10% or 1% of tumor cells with positive staining, respectively. The protein expression scoring was performed independently by two trained pathologists blinded to the clinical information, and the final score of each sample was determined by averaging the two sores made by the two pathologists.

### Statistical analysis

For gene expression in TCGA database, high and low expression of immune checkpoints and CD8 was defined with the median expression value as a cutoff. The box-and-whisker plots, heat maps, and scatter plots were generated with the gplots package and built-in R graphic functions in R/Bioconductor software. Kaplan–Meier survival curves were produced with the survfit function in the survival package, and the survival curves were compared with log-rank tests. For the T helper 1 cell (Th1)/IFNγ gene signature, we used the published Th1 signature genes from Gentleman and colleagues [70] and the IFNγ signaling pathway genes from Reactome (http://www.reactome.org; Supplementary Table 2). A Pearson T test statistic was used to analyze the relationship between B7-H3 (CD276) and CD47 expression with CD8 and the Th1/IFNγ gene signature, and T test was applied to the Spearman coefficients of correlation for tumor mutational load analysis.

### Ethics statement

The SYSUCC Ethics Committee approved this study. All patients included in our study approved and signed a written informed consent according to the policies of the SYSUCC Ethics Committee.

## Supplementary Material

Supplementary Figures

Supplementary Table 1

Supplementary Table 2
