# Supplementary material for: Identification of immunological subtypes of hepatocellular carcinoma with expression profiling of immune-modulating genes
Source: Aging (Albany NY). 2020 Jun 16;12(12):12187–205. doi: 10.18632/aging.103395 (PMC7343492; doi:10.18632/aging.103395)
Supplement: Supplementary Figures [file aging-12-103395-s003..pdf]

SUPPLEMENTARY FIGURES

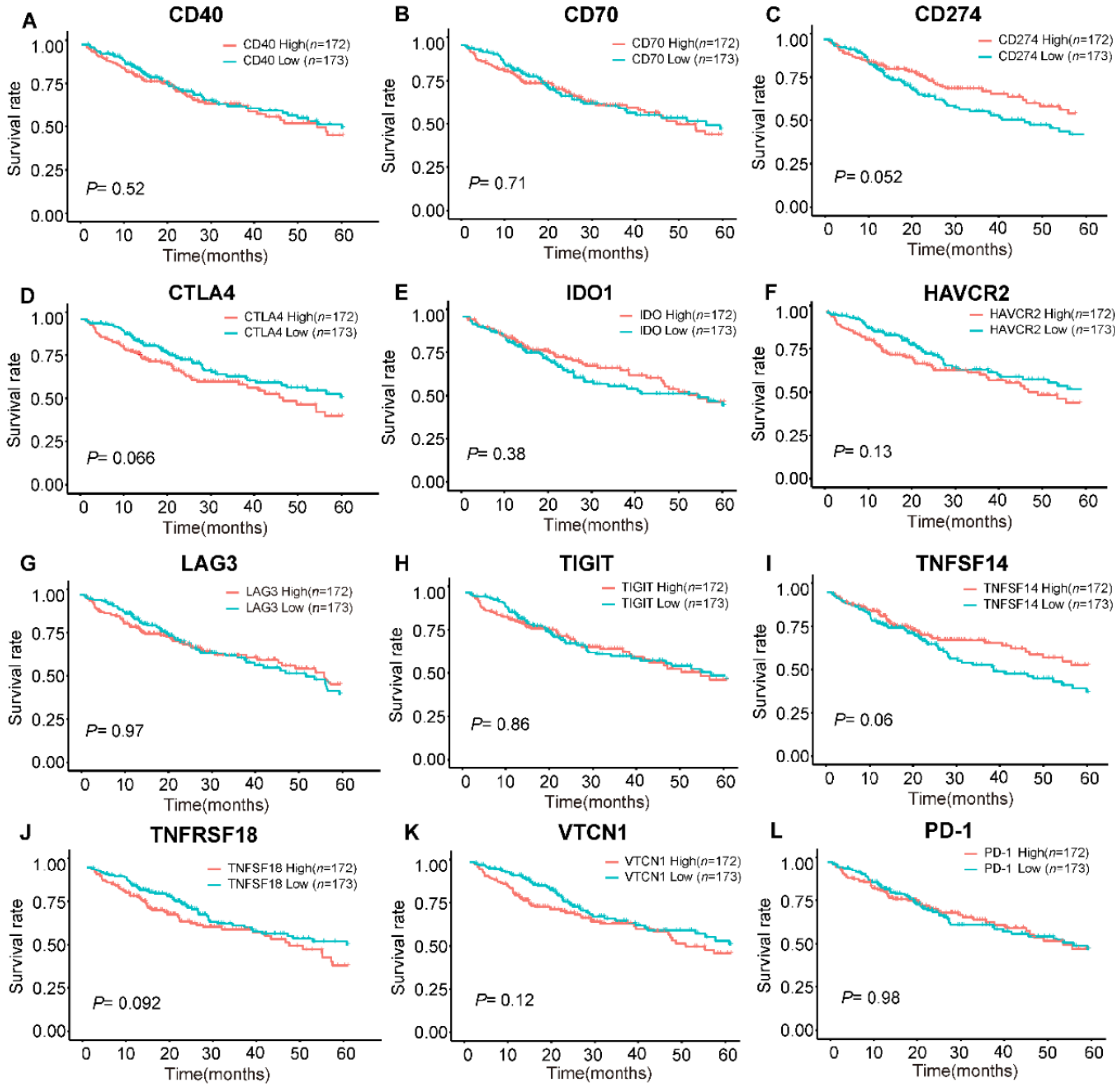

Supplementary Figure 1. Survival analysis of HCC patients based on the expression levels of adaptive immune resistance gene in TCGA HCC cohort (n = 345). (A-L) Kaplan–Meier plots of survival based on the expression of selected adaptive immune resistance markers CD40, CD70, CD274(PD-L1), CTLA4, IDO1, HAVCR2, LAG3, TIGIT, TNFSF14, TNFRSF18, VTCN1 and PD-1. The results show that these gene expressions are not associated with the survival of HCC patients (log rank test).

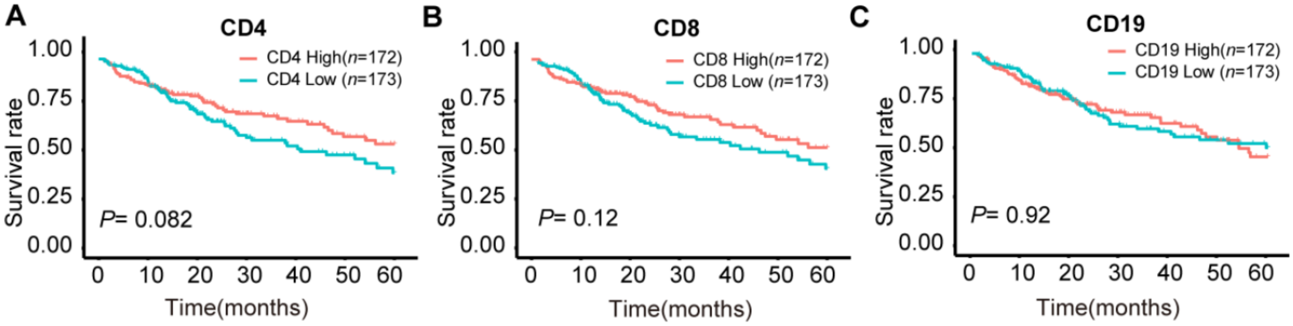

Supplementary Figure 2. Survival curves of HCC patients with high and low expressions of immune markers in TCGA HCC cohort. (**A**) Survival curves of HCC patients with high and low expression of CD4. (**B**) Survival curves of HCC patients with high and low expression of CD8. (**C**) Survival curves of HCC patients with high and low expression of CD19. The results indicate that 3 genes are not significantly associated with the survival of HCC patients log rank test.

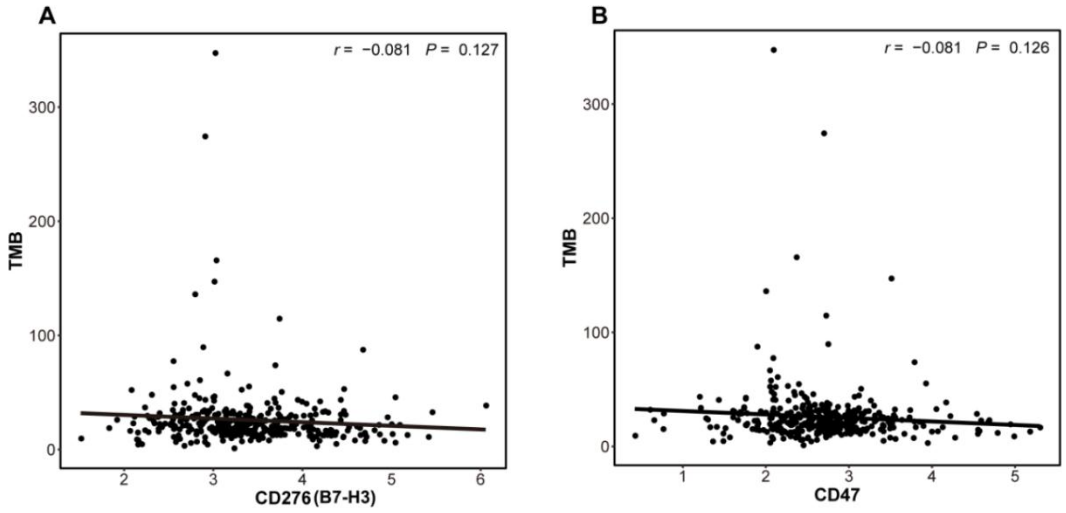

Supplementary Figure 3. The correlations between tumor mutational load (TMB) and B7-H3 or CD47 expression. (**A**) The relationship between the TMB and B7-H3 expression. (**B**) The relationship between the TMB and CD47 expression. The coefficient and the corresponding *P*-value from the Pearson's correlation test is displayed.

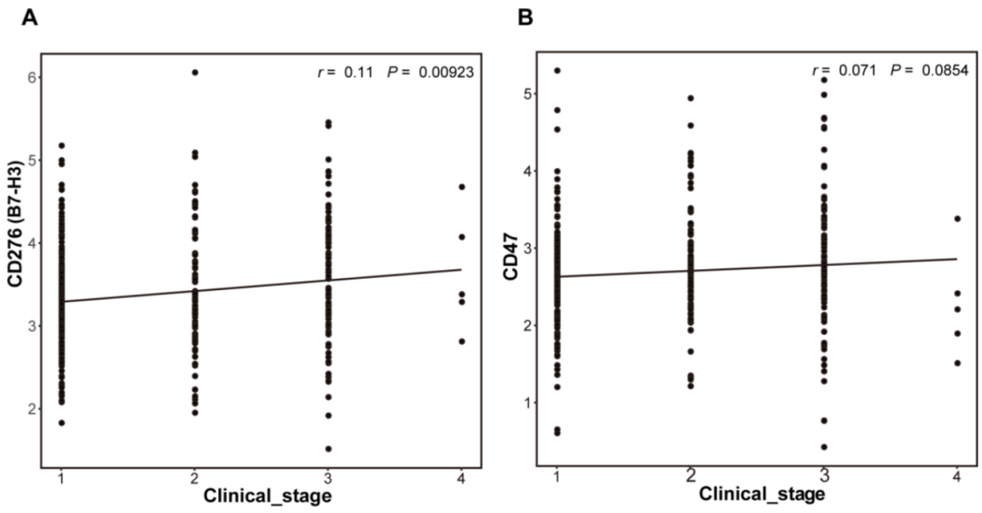

Supplementary Figure 4. The relationship between clinical stage and B7-H3 or CD47. (**A**) B7-H3 expression is significantly positively correlated with the clinical stages. (**B**) CD47 expression is marginally positively correlated with the tumor stages. The coefficient and the corresponding P-value from the Spearman's correlation test is displayed.
