# Supplementary material for: Identification of immunological subtypes of hepatocellular carcinoma with expression profiling of immune-modulating genes
Source: Aging (Albany NY). 2020 Jun 16;12(12):12187–205. doi: 10.18632/aging.103395 (PMC7343492; doi:10.18632/aging.103395)
Supplement: Supplementary Table 1 [file aging-12-103395-s002..pdf]

SUPPLEMENTARY TABLES

Supplementary Table 1. Information of antibodies of CD8, B7-H3, and CD47.

Antibody	Clone	Source	Antigen retrieval condition	Antibody dilution	Antibody incubation time
CD8	C8/144B	Cell Signaling Technology	EDTA buffer, pH9.0	1:200	18hrs
B7-H3 (CD276)	D9M2L	Cell Signaling Technology	EDTA buffer, pH9.0	1:300	18hrs
CD47	D307P	Cell Signaling Technology	EDTA buffer, pH 9.0	1:300	18hrs

Please browse Full Text version to see the data of Supplementary Table 2.

Supplementary Table 2. The correlation coefficients between Th1/IFNγ signature genes and B7-H3 or CD47.
