# Supplementary material for: Identification of immunological subtypes of hepatocellular carcinoma with expression profiling of immune-modulating genes
Source: Aging (Albany NY). 2020 Jun 16;12(12):12187–205. doi: 10.18632/aging.103395 (PMC7343492; doi:10.18632/aging.103395)
Supplement: Supplementary Table 2 [file aging-12-103395-s001..pdf]

Supplemental Table S2. The correlation coefficients between Th1/IFNγ signature genes and with B7-H3 or CD47.

Gene Symbol	B7-H3		CD47	
	Pearson's correlation coefficients	*P* value	Pearson's correlation coefficients	*P* value
GBP4	0.03	0.62	0.36	0.00
GBP5	0.19	0.00	0.28	0.00
GBP1	-0.17	0.00	0.25	0.00
STAT1	0.34	0.00	0.41	0.00
PTAFR	0.47	0.00	0.47	0.00
JAK2	0.32	0.00	0.46	0.00
JAK1	0.13	0.02	0.41	0.00
APBB2	0.10	0.06	0.26	0.00
PTPN1	0.40	0.00	0.31	0.00
CD38	0.12	0.02	0.19	0.00
OAS3	0.21	0.00	0.39	0.00
VCAM1	0.29	0.00	0.12	0.03
DGKI	0.25	0.00	0.18	0.00
CMAH	-0.04	0.50	0.09	0.09
PIAS1	0.35	0.00	0.35	0.00
IL12RB2	-0.09	0.09	0.12	0.03
CCL4	0.15	0.00	0.21	0.00
IRF3	0.31	0.00	0.28	0.00
HLA-DPA1	0.24	0.00	0.32	0.00
IFNΓ	0.13	0.01	0.22	0.00
CIITA	0.26	0.00	0.46	0.00
B2M	-0.11	0.05	0.33	0.00
FCGR1A	0.37	0.00	0.38	0.00
GBP7	-0.38	0.00	-0.03	0.61
EGFL6	0.40	0.00	0.30	0.00
CTLA4	0.22	0.00	0.24	0.00
IFNΓR1	-0.18	0.00	0.17	0.00
CD44	0.19	0.00	0.40	0.00
HLA-DQA1	0.38	0.00	0.34	0.00
DPP4	-0.22	0.00	-0.03	0.64
SP100	-0.05	0.35	0.41	0.00
IRF4	0.05	0.40	0.16	0.00
SGCB	0.17	0.00	0.23	0.00
FCGR1B	0.36	0.00	0.35	0.00
GBP6	0.15	0.01	0.24	0.00
OAS2	0.05	0.39	0.33	0.00
SUMO1	0.19	0.00	0.20	0.00
HLA-DQA2	0.28	0.00	0.27	0.00

DUSP5	0.23	0.00	0.25	0.00
ICAM1	0.26	0.00	0.39	0.00
IRF2	0.09	0.09	0.32	0.00
LTA	0.28	0.00	0.26	0.00
HLA-DPB1	0.33	0.00	0.34	0.00
LRRN3	0.34	0.00	0.22	0.00
SOCS3	0.19	0.00	0.23	0.00
HLA-DRB1	0.22	0.00	0.33	0.00
HLA-DRB5	0.11	0.04	0.26	0.00
IFNΓR2	0.59	0.00	0.38	0.00
IRF1	0.21	0.00	0.44	0.00
APOD	0.19	0.00	0.02	0.66
CSF2	0.23	0.00	0.26	0.00
DOK5	0.12	0.02	0.11	0.04
MT2A	-0.31	0.00	-0.02	0.72
IRF9	0.17	0.00	0.24	0.00
NCAM1	0.17	0.00	0.29	0.00
CAMK2D	0.35	0.00	0.21	0.00
ATP9A	0.39	0.00	0.26	0.00
IRF7	0.18	0.00	0.25	0.00
OAS1	0.00	0.93	0.24	0.00
IRF8	0.16	0.00	0.22	0.00
GGT1	0.36	0.00	0.10	0.06
CD70	0.34	0.00	0.22	0.00
HLA-A	0.08	0.14	0.36	0.00
HLA-B	-0.03	0.54	0.33	0.00
CAMK2B	-0.01	0.82	-0.01	0.90
ZBTB32	0.28	0.00	0.23	0.00
GBP2	0.10	0.06	0.38	0.00
HBEGF	0.34	0.00	0.32	0.00
IL22	0.11	0.05	0.13	0.02
CAMK2A	-0.06	0.30	0.06	0.27
IRF6	-0.14	0.01	0.06	0.24
SOCS1	0.16	0.00	0.31	0.00
BST2	-0.06	0.24	0.21	0.00
HLA-C	-0.07	0.21	0.32	0.00
OASL	-0.22	0.00	0.09	0.11
PTPN6	0.42	0.00	0.36	0.00
HLA-F	-0.06	0.26	0.29	0.00
HLA-G	0.00	0.97	0.24	0.00
IRF5	0.37	0.00	0.31	0.00
PML	0.57	0.00	0.53	0.00
SYNGR3	0.38	0.00	0.37	0.00
PTPN2	0.56	0.00	0.40	0.00

PRKCD	0.57	0.00	0.35	0.00
LRP8	0.39	0.00	0.48	0.00
BTG3	0.39	0.00	0.39	0.00
